# Synthesis and ultrastructural observation of arbutoid mycorrhizae of black truffles (*Tuber melanosporum* and *T. aestivum*)

**DOI:** 10.1007/s00572-020-00985-5

**Published:** 2020-08-24

**Authors:** Francesca Ori, Marco Leonardi, Antonella Faccio, Fabiano Sillo, Mirco Iotti, Giovanni Pacioni, Raffaella Balestrini

**Affiliations:** 1 Department of Life, Health and Environmental Sciences, University of L’Aquila, via Vetoio, Coppito 1, 67100 L’Aquila, Italy; 2 National Research Council, Institute for Sustainable Plant Protection, Viale Mattioli 25, 10125 Torino, Italy

**Keywords:** Truffle, Strawberry tree, Co-cropping, Morphotyping, TEM, Immunolabelling

## Abstract

*Arbutus unedo* (the strawberry tree) is a Mediterranean shrub which forms arbutoid mycorrhizae with a variety of Asco- and Basidiomycetes. After the discovery of the mycorrhizal symbiosis between *A. unedo* and *Tuber borchii*, in this study, arbutoid mycorrhizae were synthetized in greenhouse with *Tuber aestivum* and *Tuber melanosporum*. Six months after inoculation, both species colonized the roots of all inoculated *A. unedo* seedlings, but mature mycorrhizae were only observed after 12 months. Ultrastructure analysis of *Tuber* arbutoid mycorrhizae was described for the first time, showing, as observed in typical endosymbiosis, a rearrangement of host cells and the creation of an interface compartment with both truffle species. Immunolabelling experiments suggested that pectins are not present in the interface matrix surrounding the intracellular hyphae. Thus, the ability to establish symbiosis with *A. unedo* seems to be a common feature in the genus *Tuber*, opening up the possibility to use this plant for mycorrhization with valuable truffles. This could represent an important economic opportunity in Mediterranean areas by combining the production of truffles, edible fruits and valued honey.

**Electronic supplementary material:**

The online version of this article (10.1007/s00572-020-00985-5) contains supplementary material, which is available to authorized users.

## Introduction

Truffles are hypogeous fungi belonging to the genus *Tuber* (Ascomycota, Pezizales) that produce edible ascomata. Bonito et al. (2010) estimated that *Tuber* contains a minimum of 180 species, some of them with high commercial value. The most famous and expensive truffle species are *Tuber magnatum* Picco and *Tuber melanosporum* Vittad., better known as the Italian white truffle and the Périgord truffle, respectively. Of great interest are also other species such as *Tuber aestivum* Vittad. and *Tuber borchii* Vittad. Cultivation of these edible truffles is becoming increasingly important in their native European countries as well as outside Europe where these fungi do not naturally grow (Zambonelli et al. 2015). Valuable *Tuber* species can grow in a variety of soil and climate conditions and can establish ectomycorrhizal (ECM) relationships with a broad range of host plants. They are reported to be symbionts predominantly of woody plants and shrubs such as oaks, hazels, pines and rockrose. However, *T. aestivum*, *T. melanosporum* and *T. borchii* have also been found in symbiotic association (as mycorrhizae or endophytes) with hosts that do not form ectomycorrhizae (Selosse et al. 2004; Ouanphanivanh et al. 2008; Gryndler 2016; Lancellotti et al. 2016; Schneider-Maunoury et al. 2018, 2020). *Arbutus* species (Ericaceae) form mycorrhizal interactions (i.e. arbutoid mycorrhizae) with fungi involved in ECM associations (Molina and Trappe 1982). Several ultrastructural studies have shown that arbutoid mycorrhizae are very similar to ectomycorrhizae with the exception of the intracellular penetration. As in the ectomycorrhizae, arbutoid associations produce an intercellular Hartig net, usually restricted to the outer layer of root cells, and a fungal sheath can be present although with a different thickness depending on the fungus involved in the association (Massicotte et al. 1993). As the main difference, in arbutoid mycorrhizae, fungal hyphae colonize the rhizodermal cells and fill them with hyphal coils (Massicotte et al. 1993). Kennedy et al. (2012) identified two *Tuber* species as fungal partners in arbutoid mycorrhizae formed with *Arbutus menziesii* Pursh, while Lancellotti et al. (2014) and Plácito et al. (2018) described arbutoid mycorrhizae between *T. borchii* and *Arbutus unedo* L. either recovered from the wild or synthetized in greenhouse. However, it needs to be demonstrated that this plant can be successfully used for large-scale programmes of truffle mycorrhization. *Arbutus unedo*, the strawberry tree, is a native Mediterranean species, naturally spread along the Atlantic coasts of some European countries including Ireland and France (Santiso et al. 2015). Since it responds well to the conditions of Mediterranean summers, such as high temperature, low humidity and water stress, it is also considered an alternative for revegetation and restoration projects in the Mediterranean basin (Navarro et al. 2007a). Besides its use in gardening (Navarro et al. 2007b), *A. unedo* is used in tannery and medicine, and its edible fruits can be consumed fresh or in processed products like alcoholic beverages, jams, jellies and compotes (Lim 2012; Morgado et al. 2018).

While the anatomy and ultrastructure of *A. unedo*-*Laccaria amethystina* Cooke arbutoid mycorrhizae have been already reported (Münzenberger et al. 1992) as well as the description of the mycorrhizae that developed between *A. unedo* and an unknown ascomycete (Fusconi and Bonfante-Fasolo 1984), at our knowledge, no information has been reported on the ultrastructural features of *A. unedo* roots colonized by a *Tuber* species. Our hypothesis was that the capacity of establishing symbiosis with *Arbutus* is a common feature in the genus *Tuber* and that the truffle arbutoid mycorrhizae have different features from the corresponding ectomycorrhizae, a difference that can be ascribed to the plant species involved. Interestingly, Fusconi and Bonfante-Fasolo (1984) suggested that arbutoid mycorrhizal structure should be considered a subtype of the ericoid mycorrhizal type. This paper reports on a morphological analysis of arbutoid mycorrhizae of *A. unedo* synthetized in pots using two different truffle species, i.e. *T. aestivum* and *T. melanosporum*. In addition, electron microscopy observations allowed us to provide a detailed description of the ultrastructural features in the two mycorrhizal types.

## Materials and methods

### Plant material and inoculum production

Seeds of *A. unedo* were collected from a single plant, sited in a private garden in Roseto degli Abruzzi (Italy). Seeds were surface sterilized in 1% sodium hypochlorite solution for 1 h and then rinsed with tap water. Seedlings were grown in a sterile peat moss/vermiculite mix (1:1) for about 5 weeks before inoculation (3–5 true leaves present). *Arbutus* seedlings were inoculated with spores of *T. aestivum* and *T. melanosporum*. The spore suspensions were obtained by crushing fresh ascomata with a Waring blender 7011 (18,000 rpm) and suspending them in sterile water. Ascomata were previously brushed under tap water and surface flame sterilized for a few seconds. Twenty seedlings (10 for each truffle species) were transplanted into plastic pots with a mix of vermiculite (50%), river sand (40%) and peat moss (10%) and then inoculated with an aliquot of about 1 × 10^6^ spores, injected close to the roots of each plant. Inocula were prepared measuring spore density of the suspension by a haemocytometer. Ten seedlings were not inoculated and used as controls. The plants were grown at 22 ± 2 °C, 80% relative humidity and 14-h photoperiod and watered twice a week.

### Evaluation of root colonization and molecular analyses

Root colonization was evaluated 6 and 12 months after inoculation. Roots were washed in sterile water and examined under a stereomicroscope equipped with a camera (Leica). Five root fragments for each plant (4–8 cm length) were examined to evaluate root colonization by *T. aestivum* and *T. melanosporum*. After collection of the root tips at the first time point (6th month), all seedlings were kept in the same pot and soil and grown for another 6 months. The colonization degree was measured by counting the number of infected and uninfected tips, and the result was expressed as a percentage. Morpho-anatomy of mycorrhizae was described following Agerer (1995). Three mycorrhizal tips for each *Tuber* species and seedling were stored in FAA (37% formaldehyde:70% ethanol/acetic acid, 5:90:5) at 4 °C pending morphometric analysis. Dimensions of 25 mycorrhizal tips (unramified ends) were measured for each species and time point (6 and 12 months).

One to three colonized tips per seedling were stored in sterile water at − 80 °C for molecular characterization. Confirmation of fungal identity was performed by applying species-specific primers of *T. aestivum* (Mello et al. 2002) and *T. melanosporum* (Rubini et al. 1998). Amplifications were carried out by applying the direct PCR technique (Iotti and Zambonelli 2006) according to which a microscopic mantle fragment is used as PCR target without prior DNA extraction.

### Anatomy and ultrastructure

#### Fixation, embedding and microscope observations

Anatomy and ultrastructure of truffle arbutoid mycorrhizae were studied on longitudinal sections. Fresh mycorrhizae were fixed in 2.5% (v/v) glutaraldehyde in 10 mM Na-phosphate buffer (pH 7.2) for 2 h at room temperature and then overnight at 4 °C. After rinsing with the same buffer, they were dehydrated in an ethanol series (30, 50, 70 and 90% for 15 min each step, and 2 times 100% for 20 min each step) at room temperature. Dehydrated ectomycorrhizae were infiltrated in 3:l (v/v) absolute ethanol/London Resin White resin (Multilab Supplies) (E/LRW) for 1 h, 1:1 E/LRW for 1 h, 1:3 E/LRW for 1 h and 100% London Resin white overnight at 4 °C according to Balestrini et al. (1996). The mycorrhizae were then embedded in gelatine capsules and polymerized for 24 h at 60 °C. Semi-thin sections (2.25 μm) were cut using an ultramicrotome and stained with 1% toluidine blue in 1% Na borate. Mycorrhizal anatomy and mantle thickness were analysed under a microscope equipped with a camera (Leica). Ninety measurements (30 × 3 mycorrhizal tips) were taken for each species, time point (6 and 12 months) and root tip position (proximal or distal part of tips).

For the ultrastructural observations, after the preparation of semi-thin sections (1 μm) stained with 1% toluidine blue, ultra-thin sections (0.05–0.07 μm) were cut and poststained with Uranyl Acetate Substituted (Agar Scientific, Stansted UK) and lead citrate before observation with Philips (Eindhoven, The Netherlands) CM 10 transmission electron microscope operated at 60–80 kV.

#### Immunolabelling

Ultra-thin sections were incubated for 15 min in normal goat serum diluted 1:30 in 0.05 M Tris-HC1 buffer with 0.9% NaCl (TBS, pH 7.6) and 0.2% BSA and treated overnight with the different monoclonal antibodies (1:1 dilution) to localize pectins (JIM5, LM19, JIM7; CarboSource, https://www.ccrc.uga.edu/~carbosource/CSS_mabs7-07.html). After washing, they were incubated for 1 h with 15 nm of colloidal gold goat-anti-rat IgG complex (BBI Solutions, Crumlin UK) containing 1% BSA (diluted 1:20 in TBS). Thin sections were then post-stained as described above (ultrastructural observations). Labelling specificity was determined by replacing the primary antibody with the buffer.

### Statistical analysis

A two-way ANOVA with robust estimators (number of bootstrap samples = 5000) procedure was used to compare mantle thickness at different positions and times after inoculation for both *T. melanosporum* and *T. aestivum*. A Wilcoxon signed-rank test was used to assess significant differences between mantle thickness at different time points for each species, as well as to assess differences in mycorrhizal diameters and length. For all tests, differences were considered statistically significant with a probability level of *P* ≤ 0.05. In order to assess the effect of time and species in terms of number of observed mycorrhizae, a two-way ANOVA with robust estimators (number of bootstrap samples = 5000) was used, and mean separation was performed using the Tukey HSD test, adopting a probability level of *P* ≤ 0.05. All tests were conducted by using R (basic and *WRS2* packages).

## Results

### Root colonization and morphology

**Fig. 1:**
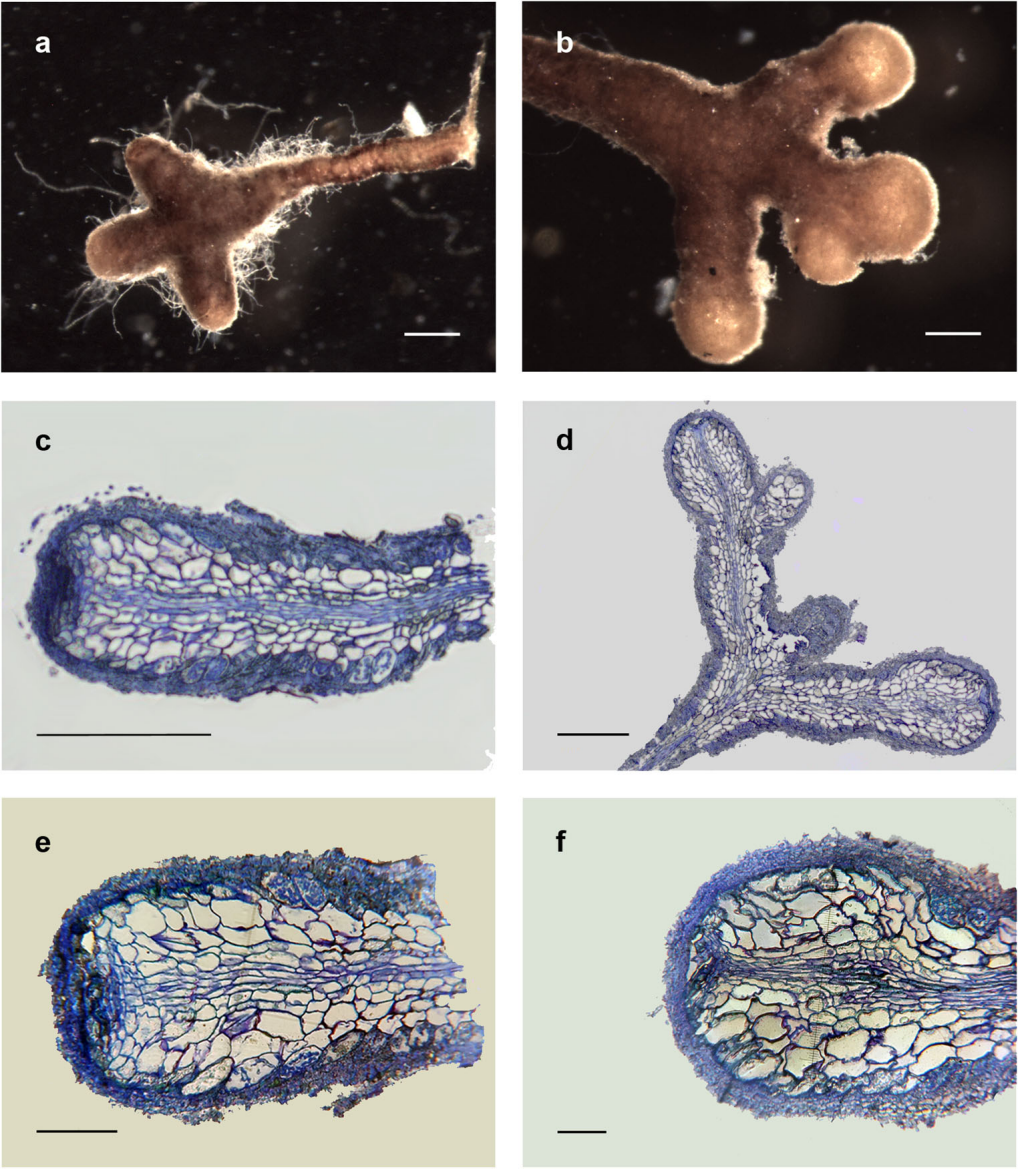
Arbutoid mycorrhizae of *T. aestivum* (**a**, **c**, **e**) and *T. melanosporum* (**b**, **d**, **f**) 12 months after inoculation. Bars correspond to 250 μm for whole mycorrhizae (**a**, **b**) and 200 μm (**c**, **d**) or 50 μm (**e**, **f**) for longitudinal sections

Six months after inoculation, all seedlings were found to be colonized by the inoculated truffle species with a mean value of 27 ± 4% and 24 ± 5% for *T. aestivum* and *T. melanosporum*, respectively. Root colonization increased to 57 ± 17% and 46 ± 16% after further 6 months of seedling growth. Significant effects of time, but not species, was determined on mycorrhizal colonization, which was significantly higher (*P* ≤ 0.05) for both species at 12 months compared with 6 months (Fig. 1S). All PCRs confirmed the identity of truffle mycorrhizae by producing species-specific amplicons of 402 bp and 447 bp for *T. aestivum* and *T. melanosporum*, respectively (data not shown). No other ectomycorrhizal fungi were found to colonize the roots of any *A. unedo* seedlings.

**Fig. 2:**
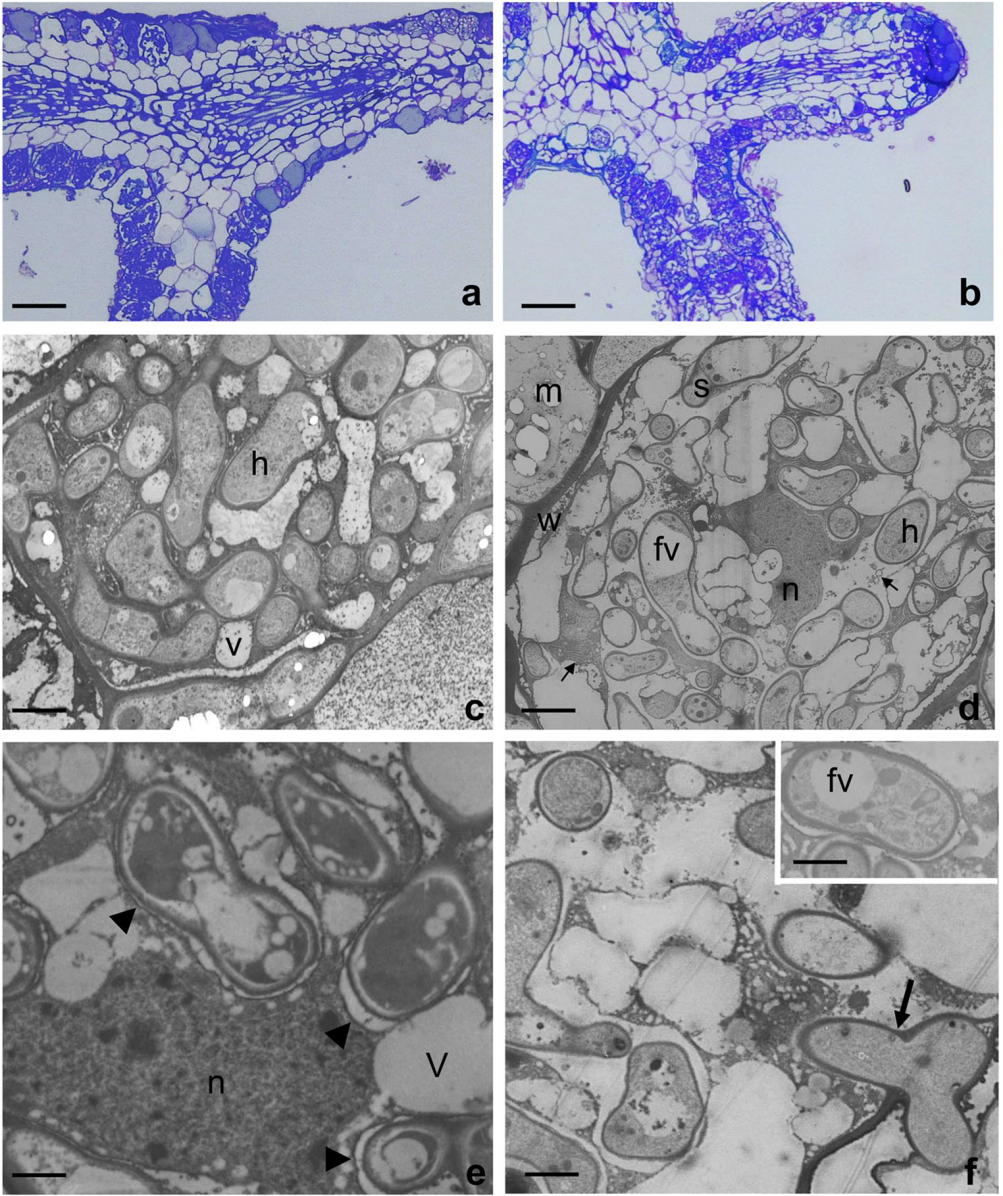
Ultrastructural features of 6-month-old arbutoid mycorrhizae between *A. unedo* and *T. melanosporum* (**a**, **c**, **e**) and *T. aestivum* (**b**, **d**, **f**), respectively. **a**–**b** Longitudinal section of *T. melanosporum* (**a**) and *T. aestivum* (**b**) arbutoid mycorrhizae showing a thin mantle. Mantle hyphae penetrate between rhizodermal cells to form the Hartig net (arrows) and colonize rhizodermal cells (asterisks). Hyphal chains forming the Hartig net are particularly evident in *T. melanosporum* mycorrhiza (**a**), while they are less evident in the same region of *T. borchii* ones (**b**). Bars correspond to 40 μm (**a**) and 50 μm (**b**). **c** Transmission electron picture showing a rhizodermal cell containing fungal hyphae (**h**) in a mature *T. melanosporum* mycorrhiza. Small vacuoles (v) are evident inside the cells. Bar corresponds to 2 μm. **d** Transmission electron picture of a *T. aestivum* colonized host cell. Membranous systems are evident in the host cell cytoplasm as well as small vesicles (arrows) and an irregular shaped nucleus (n) positioned at the centre of the cell. Hyphae show septa with Woronin bodies (s) as well as vacuoles (fv). m, mantle; w, host cell wall. Bar corresponds to 2.5 μm. **e** Magnification of *T. melanosporum* intracellular hyphae. An electron-transparent interface space, delimited by the fungal cell wall and the host plasmalemma, is evident around the fungal hyphae (arrowheads). n, plant nucleus. Bar corresponds to 1 μm. **f** Magnification of a *T. aestivum* colonized cell. A penetration point is evident (arrow). The interface space is evident around a *T. aestivum* intracellular hypha. Bars correspond to 1.8 μm (f) and 1 μm (inset)

Mycorrhizae analysed 6 months after inoculation had a smooth mantle without cystidia regardless the truffle species. Rhizodermal cells and Hartig net were visible in transparency under fungal mantle forming a brown reticulum. *Tuber melanosporum* mycorrhizae had a higher number of cruciform ramifications (40%) than *T. aestivum* (20%). Most tips of *T. aestivum* were clavate (Fig. 2Sa), while those of *T. melanosporum* were mostly tapering without an evident mantle at the apex (Fig. 2Sb).

*Tuber aestivum* mycorrhizae were ochre in colour, with tips 435 ± 83 μm long and 195 ± 35 μm wide. *Tuber melanosporum* mycorrhizae were ochre-brown in colour, with tips 632 ± 182 μm long and 158 ± 24 μm wide. Twelve months after inoculation, mycorrhizae of both truffle species were mostly cruciform (> 90%), clavate and ochre (distally) to brown (proximally), with abundant (*T. aestivum*) or sparse (*T. melanosporum*) cystidia (Fig. 1a, b). The size of tips increased in both *T. melanosporum* (729 ± 195 μm × 218 ± 27 μm) and *T. aestivum* (804 ± 194 μm × 219 ± 30 μm). Diameters and length of tips at 12 months were significantly different (*P* ≤ 0.05) from those at 6 months for *T. aestivum*, while only tip diameter significantly differed (*P* ≤ 0.05) over time for *T. melanosporum* (Fig. 3S).

### Anatomy and ultrastructure

Microscopical observations showed that mycorrhizae of both species had a pseudoparenchymatous mantle with angular (type L) or epidermoid (type M) cells for *T. aestivum* and *T. melanosporum*, respectively. The semi-thin longitudinal sections of 6-month-old mycorrhizae showed the lumen of the rhizodermal cells almost completely colonized by hyphal coils, which became less frequent or absent towards the apex of tips. Colonized cells were inflated with the typical “fish bone” appearance as in ectomycorrhizae (Smith and Read 2008; Balestrini and Kottke 2017). Hyphae of both species never colonized the cortical cell layers by Hartig net or hyphal coils. *Tuber aestivum* mycorrhizal tips showed a 3–4 cell-layer mantle thicker (Table 1, Figs. 2Sc, e), while only 1 to 3 layers were observed in *T. melanosporum* mycorrhizal tips (Figs. 2Sd, f). Most of *T. melanosporum* mycorrhizae did not show any type of fungal structure (mantle, Hartig net or hyphal coils) at the root apex or, rarely, a one-layered mantle (Table 1).

**Table 1:** Mantle thickness of *T. aestivum* and *T. melanosporum* mycorrhizae, 6 and 12 months after inoculation, at the proximal and distal parts of tips (*n* = 90)

Time after inoculation	Mantle thickness (μm)			
	*T. aestivum*		*T. melanosporum*	
	Proximal	Distal	Proximal	Distal^*^
6 months	14.3 ± 3.2	10.7 ± 2.9a	15.7 ± 4.4	7.4 ± 2.3a
12 months	19.2 ± 0.2	17.1 ± 4.7b	19.1 ± 0.2	22.2 ± 5.2b

^*^T. melanosporum mycorrhizae without a mantle in the distal part of tips were excluded from the measurement; different letters on the same column indicate significant differences for *P* > 0.05

Twelve months after inoculation, mycorrhizae showed a 3–4 layers continuous mantle regardless the truffle species (Fig. 1c–f). Hartig net and hyphal coils were also spread at the root apex although the level of intra-cellular colonization was less evident than that present in the older regions of mycorrhizae. Mantle thickness consistently increased in any part of mycorrhizal tips regardless of the truffle species (Table 1). The two-way ANOVA with robust estimators confirmed the effect of time and position on mantle thickness for both species (*P* ≤ 0.05). Mantle thickness at 6 and 12 months was significantly different in the distal parts (*P* ≤ 0.05) for both species, but not in the proximal parts (*P* ≥ 0.05) (Table 1 and Fig. 4S).

Ultrastructural observations were performed on 6-month-old mycorrhizal tips, mostly to verify the intracellular colonization (Fig. 2). In addition to the development of a Hartig net (Fig. 2a, b), hyphae can penetrate rhizodermal cells, growing and branching inside them to form the coil structures typical of ecto-endomycorrhizae (Fig. 2a, b). In this area, in agreement with previous observations (Fusconi and Bonfante-Fasolo 1984), host cells show the presence of vacuoles with electron-dense material, probably tannins (Fig. 2c). It is worth noting that, during cell colonization, the host vacuoles are split in smaller ones by the intracellular invasion by the fungal hyphae (Fig. 2c). Vesicles are evident in the host cytoplasm, mainly in *T. aestivum* mycorrhizae (Fig. 2d). The plant nucleus, as in other endomycorrhizae, has an irregular shape and can occupy a central position (Fig. 2d). Intracellular hyphae also showed a living and active cytoplasm, with small vacuoles, which contain electron-dense globular bodies (probably protein bodies), vesicles and mitochondria (Fig. 2). The walls of the intracellular hyphae are still formed by an electron-transparent inner layer and an electron-dense outer one, as typical for the truffle cell wall (Balestrini et al. 2012). The intracellular interface between the fungal hyphae and the host cells is characterized by an electron-transparent space where a fibrillar material can be present (Fig. 2). Differences in the interface features between the two fungal species, i.e. a more evident electron-dense loose material around the *T. melanosporum* hyphae, were also observed (Fig. 2c, e vs Fig. 2d, f). Pectins were not detected by the JIM5, JIM7 and LM19 antibodies on the interface material surrounding the truffle hyphae, independently from the considered fungal species.

## Discussion

The present study shows the possibility to obtain mature arbutoid mycorrhizae between *T. aestivum* and *T. melanosporum* with *A. unedo* in controlled conditions and analysed the development of mycorrhizal symbiosis over a period of 12 months. Additionally, ultrastructural description of the mycorrhizae after 6 months from inoculation was performed. The description of arbutoid mycorrhizae was already reported in two *Arbutus* species, *A. unedo* and *A. menziesii*, mainly in combination with basidiomycetes (Massicotte et al. 1993; Navarro et al. 2009, 2011; Gomes et al. 2016; Kühdorf et al. 2016), while only a few reports focused on ascomycetes such as truffle.

Arbutoid mycorrhizae formed by *T. borchii* were described by Lancellotti et al. (2014) who observed the typical characteristics of *T. borchii* ectomycorrhizae but with an intracellular colonization. *Tuber aestivum* and *T. melanosporum* arbutoid mycorrhizae are also typical arbutoid mycorrhizae with anatomical features characteristic of the respective ectomycorrhizae. Apart from the intracellular colonization, the only difference seems to be the longer time required to form fully developed arbutoid mycorrhizae with respect to that required to produce their ectomycorrhizae in greenhouse conditions (usually 3 to 6 months after inoculation). In particular, both species took more than 6 months to form cruciform branched mycorrhizae with a completely formed mantle and typical cystidia. The symbiosis development seems to be particularly slow for *T. melanosporum*, which has difficulties to follow the elongation of the young root tips. Possibly, *T. melanosporum* has a lower affinity for *A. unedo* than *T. aestivum* or *T. borchii*. However, further mycorrhization trials in different growth conditions should be carried out to confirm this observation.

Mature *T. melanosporum* and *T. aestivum* arbutoid mycorrhizae showed tip dimensions similar to those reported for *T. borchii*-*A. unedo* association, and for the corresponding ectomycorrhizae, with the exception of the tip diameter which seem to be slightly greater in *T. borchii* arbutoid mycorrhizae and pine ectomycorrhizae (Zambonelli et al., 1993, 1995; Granetti, 1995; Lancellotti et al., 2014). In agreement with previous observation on arbutoid mycorrhizae formed by *Lactarius deliciosus* (Gomes et al. 2016), *Pisolithus tinctorius* or *Piloderma bicolor* (Massicotte et al. 1993), a thick mantle is also formed by *Tuber* species (see also Lancellotti et al. 2014, for *T. borchii*). This is a different feature with respect to arbutoid mycorrhizae synthetized between *A. unedo* and an unknown ascomycete after Fusconi and Bonfante-Fasolo (1984).

Although in ectomycorrhizae the fungus usually remains apoplastic (Balestrini and Kottke 2017), the occurrence of truffle hyphae penetrating inside the host cells has been already reported for orchid roots (Selosse et al., 2004) and in vitro colonization systems with *T. borchii* (Montanini et al. 2003; Miozzi et al. 2005), which has been described to penetrate and colonize some senescent host cells in association with *Cistus incanus* (Miozzi et al. 2005). Pacioni et al. (2014) also reported the in vitro development and maintenance of endomycorrhizal associations of two *Tuber* species with transformed roots of *C. incanus*, confirming the capacity for these fungi to penetrate the host cells, at least under suitable conditions. However, histological observations of *A. unedo* mycorrhizae showed a different scenario, with the rhizodermal cells completely filled with the mycorrhizal fungus (Giovannetti et al. 1990). The same picture has been found in the truffle arbutoid mycorrhizae, where intracellular hyphal complexes were observed in rhizodermal cells with both *T. aestivum* and *T. melanosporum*. It is worth noting that arbutoid mycorrhizae are formed mainly by ECM fungi (Massicotte et al. 1993; Trevor et al. 2001). Although morphological observations on several ecto-endomycorrhizae were performed, ultrastructural analyses were not largely investigated. Our observations confirm previous studies (Fusconi and Bonfante-Fasolo 1984), showing a rearrangement of host cells and the creation of an interface compartment as observed for typical endosymbioses. Thus, describing the plant/fungus interface of arbutoid mycorrhizae, in addition to the typical ECM interface, where the plant and fungal cell wall are in direct contact, an additional interface compartment typical of endomycorrhizae is formed around the intracellular hyphae (Fusconi and Bonfante-Fasolo 1984). Our immunolabelling experiments with antibodies recognizing pectins at a different methylation degree suggest that this plant cell wall compound is not present in the interface matrix surrounding the intracellular hyphae. Pectins were found to be present in the interface compartment of intracellular mycorrhizal symbiosis (Balestrini and Bonfante 2014). However, the nature of the interface compartment is also related to the peripheral cell wall composition (Balestrini et al. 1996). A lack or a poor reaction with JIM5 and JIM7 antibodies was already observed in Ericaceae (Carney and Ashford 2002), suggesting negligible amounts of unesterified pectin and pectin with a low methyl-esterification degree. Here, a labelling was only sporadically observed on the peripheral cell wall using JIM5 as a probe (results not shown). In orchid mycorrhizae, the absence of epitopes recognized by JIM5 was reported at the interface between *Limodorum abortivum* and its *Russula* symbiont, which is the dominant fungal partner of *L. abortivum*, while pectin was found in the region surrounding the intracellular hyphae of *Ceratobasidium* (Paduano et al. 2011), suggesting that plant responses towards distinct mycorrhizal fungal partners can vary. Interestingly, truffles, as the fungi belonging to the genus *Russula*, are well known for their ECM phenotype on tree species. It is also worth noting that in truffle ectomycorrhizae, a localized degradation of pectin seems to occur during fungal growth through the middle lamella, in agreement with the expression of fungal genes acting on these polysaccharides, as observed in the *T. melanosporum*-*Corylus avellana* association (Sillo et al. 2016). Thus, further analyses are needed to improve our observations and to characterize the interfaces occurring in ecto-endomycorrhizae, i.e. using probes for different cell wall components as well as performing molecular analyses on cell wall degrading enzyme expression in ecto-endomycorrhizae. In addition, the possibility to have both ECM and arbutoid mycorrhizae with the same fungal partner is a great opportunity to study the variability in the symbiosis establishment and functioning.

However, although the establishment of symbiosis with *A. unedo* seems to be a common feature in the genus *Tuber*, it is necessary to verify whether the symbiosis with *T. aestivum* and *T. melanosporum* is common in natural conditions. Soil composition should not be the limiting factor. *Tuber aestivum* can grow in a wide range of soils (Robin et al. 2016), while *T. melanosporum* prefers dolomitic limestones and calcareous sandstones with a pH > 7 (Jaillard et al. 2016). For its part, *A. unedo* grows in different soil types, and local ecotypes have been found on basic and calcareous soils throughout the Mediterranean basin (Başlar et al. 2002; Torres et al. 2002). However, the ability of *T. aestivum* and *T. melanosporum* to establish symbiosis with *A. unedo* increases the number of host plant/truffle combinations suitable for truffle mycorrhization. They are the most cultivated truffle species in the world, and their distribution is increasing from year to year as “monocrop”. The possibility of using host plants that provide secondary products (honey and fruits in the case of *A. unedo*) in addition to truffles can be considered as an improvement to the traditional concept of truffle cultivation. Multi-cropping of economically valuable plants and truffles represents an alternative for rural and disadvantaged economies by providing farmers a multiple source of income (Benucci et al. 2012; Álvarez-Lafuente et al. 2018).

It is well known that mycorrhizal fungi improve plant fitness and protect them from environmental stresses. A role for ecto-endomycorrhizal fungi, such as the desert truffle *Terfezia claveryi* Chatin, in reducing negative effects of drought stress on *Helianthemum almeriense*, through physiological and nutritional mechanisms, has been suggested before (Morte et al. 2000). This point could be further investigated also for truffle arbutoid symbiosis, to verify its potential as multipurpose forestations in Mediterranean semi-arid regions. *Tuber melanosporum* and *T. aestivum* are typical Mediterranean truffles, and they are also adapted to warm climate although *T. melanosporum* does not tolerate extreme summer drought (Kagan-Zur et al. 2012; Zambonelli et al. 2014; Le Tacon 2016). The production of *A. unedo* plants inoculated with selected truffle strains tolerant to drought and high temperatures can represent an efficient approach to successfully cultivate truffles in semi-arid climates (Leonardi et al. 2017). As highlighted above, besides the applied aspects, the availability of ectomycorrhizae and ecto-endomycorrhizae with the same fungal species represents an important point to improve our knowledge on the mycorrhizal toolkit common to different symbiotic strategies.

## Electronic supplementary material

Plot representing percentage of mycorrhizae at different times (6 m = 6 months, 12 m = 12 months) in *T. aestivum* and *T. melanosporum* (n = 10). Data from *T. aestivum* are plotted in blue, while data from *T. melanosporum* are in red. Letters are plotted according to outcomes of the Tukey HDS test (EPS 18735 kb)

Arbutoid mycorrhizae of *T. aestivum* (a, c, e) and *T. melanosporum* (b, d, f) six months after inoculation. Bars correspond to 250 μm for whole mycorrhizae (a, b) and 200 μm (c, d) or 50 μm (e, f) for longitudinal sections (TIF 6270 kb)

Boxplots representing data of diameter and length of mycorrhizal tips at different times (6 m = 6 months, 12 m = 12 months) in *T. aestivum* and *T. melanosporum* (n = 25). Data from *T. aestivum* are plotted in blue, while data from *T. melanosporum* are in red. Letters are plotted according to outcomes of Wilcoxon signed-rank test (EPS 18953 kb)

Boxplots representing data of mantle thickness at different times (6 m = 6 months, 12 m = 12 months) and in different parts of mycorrhizal tips (proximal and distal) in *T. aestivum* and *T. melanosporum* (n = 90). Data from *T. aestivum* are plotted in blue, while data from *T. melanosporum* are in red. Letters are plotted according to outcomes of Wilcoxon signed-rank test (EPS 19429 kb)
