# Supplementary figures and images for: Synthesis and ultrastructural observation of arbutoid mycorrhizae of black truffles (Tuber melanosporum and T. aestivum)
Source: Mycorrhiza. 2020 Aug 24;30(6):715–23. doi: 10.1007/s00572-020-00985-5 (PMC7591440; doi:10.1007/s00572-020-00985-5)

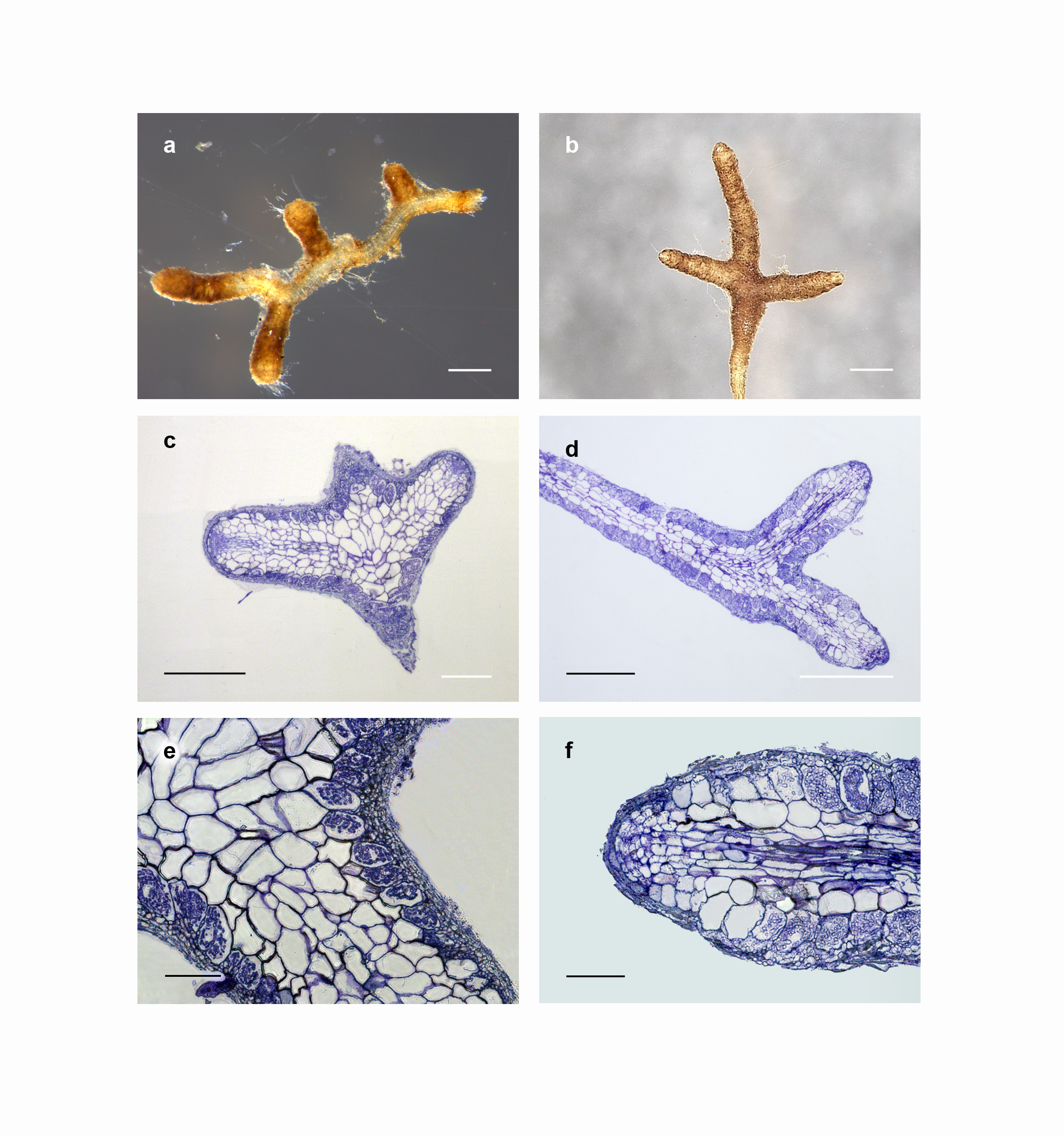

Supplement: Supplementary file 2 — Arbutoid mycorrhizae of T. aestivum (a, c, e) and T. melanosporum (b, d, f) six months after inoculation. Bars correspond to 250 μm for whole mycorrhizae (a, b) and 200 μm (c, d) or 50 μm (e, f) for longitudinal sections (TIF 6270 kb) [file 572_2020_985_MOESM2_ESM.tif]
